# DNA Methylation Differences Between Zona Pellucida-Bound and Manually Selected Spermatozoa Are Associated With Autism Susceptibility

**DOI:** 10.3389/fendo.2021.774260

**Published:** 2021-11-09

**Authors:** Longda Wang, Mengxiang Chen, Gaofeng Yan, Shuhua Zhao

**Affiliations:** Department of Reproduction and Genetics, First Affiliated Hospital of Kunming Medical University, Kunming, China

**Keywords:** sperm, DNA methylation, autism, zona pellucida binding assay, ICSI

## Abstract

Children conceived through intracytoplasmic sperm injection (ICSI) have been reported to have a higher risk of many abnormalities and disorders, including autism and intellectual disability, which may be due to bypassing of the natural sperm selection process during ICSI. Zona pellucida (ZP)-bound spermatozoa (ZPBS) have normal morphology and nuclear DNA. Using these spermatozoa for ICSI results in better outcomes compared with conventional ICSI. However, differences besides morphology that exist between sperm selected by ZP and by an embryologist and whether these differences affect the risk of autism in offspring after ICSI are unclear. To explore these questions, we compared genome-wide DNA methylation profiles between ZPBS and manually selected spermatozoa (MSS)using single-cell bisulfite sequencing. Global DNA methylation levels were significantly lower in ZPBS than in MSS. Using gene ontology (GO) analysis, genes overlapping differentially methylated regions (DMRs) were enriched in biological processes involving neurogenesis. Furthermore, we found that 47.8% of autism candidate genes were associated with DMRs, compared with 37.1% of matched background genes (*P*<0.001). This was mainly because of the high proportion of autism candidate genes with bivalent chromatin structure. In conclusion, bivalent chromatin structure results in large differences in the methylation of autism genes between MSS and ZPBS. ICSI using MSS, which increases the risk of methylation mutations compared with ZPBS, may lead to a higher risk of autism in offspring.

## Introduction

Infertility is one of the most common health disorders globally, affecting approximately 15% of the reproductive-aged population, with male infertility accounting for 30-50% of cases (1). A large proportion of these patients undergo intracytoplasmic sperm injection (ICSI), which involves the injection of a single spermatozoon into an oocyte cytoplasm using a glass micropipette. ICSI is currently used far beyond its original application to overcome the most severe forms of male infertility. In the United States, the use of ICSI for all non-male factor infertility cases increased from 15.4% in 1996 to 66.9% in 2012 (2). Although ICSI may benefit couples by increasing the fertilization rate and decreasing the fertilization failure rate, there are concerns that the indiscriminate use of ICSI may lead to adverse health consequences for the offspring (3). For example, the risks of congenital malformations, epigenetic disorders, chromosomal abnormalities, subfertility, cancer, delayed psychological and neurological development, and impaired cardiometabolic profiles have been shown to be greater in infants born as a result of ICSI than in naturally conceived children (3).

A meta-analysis has reported a higher incidence of intellectual disability and autism, a set of heterogeneous neurodevelopmental conditions characterized by early-onset difficulties in social communication and unusually restricted, repetitive behavior and interests, in children conceived with ICSI (4). Importantly, the association between autism and ICSI is stronger among children conceived with ICSI for non-male factors, suggesting that autism may be associated with factors besides male infertility factors (5).During ICSI, the direct injection of a single spermatozoon into the egg bypasses the processes of natural sperm selection that occur during normal fertilization, which may increase the risk of transmission of genetic or epigenetic mutations in spermatozoa that are naturally unable to bind and enter eggs.

However, evidence supporting the link between ICSI sperm selection and autism is lacking. There is evidence showing the superiority of spermatozoa naturally selected by zona pellucida (ZP), a transparent, extracellular matrix composed of defined glycoproteins and containing receptors that are crucial for sperm-egg binding, sperm selection, and fertilization (6). Compared with unselected sperm, ZP-bound sperm (ZPBS) have significantly higher rates of normal morphology and nuclear DNA (7, 8). ICSI using ZPBS results in improved embryo quality and higher implantation rate than ICSI using spermatozoa manually selected by embryologists (9–11). These differences may be due to the high heterogeneity of spermatozoa even from the same ejaculate. Because of variations in motility and morphology, only 14% of spermatozoa from normozoospermic men can bind to ZP (12). In addition to morphological and functional heterogeneity, sperm heterogeneity is also found at the DNA level. Breuss et al. (13)reported sperm mosaicism using deep whole-genome sequencing and identified single nucleotide, structural, and short tandem repeat variants. Notably, the quantification of sperm mosaicism can stratify the risk of autism recurrence due to *de novo* mutations, and novel mosaic risk variants are able to be transmitted to offspring.

Compared with nucleotide mutations, DNA methylation alterations are more like to occur after exposure to risk factors. Jenkins et al. (14) reported intra-sample heterogeneity of sperm DNA methylation by comparing methylation profiles between high- and low-quality sperm separated using gradient separation. However, even among “high-quality” sperm, the proportion of functionally normal sperm is low (12, 15). Whether DNA methylation differences exist between naturally selected (functionally normal) sperm and unselected sperm or embryologist-selected sperm for ICSI, and whether these differences are related to male infertility and/or the health of children conceived after ICSI, have not been determined. To answer these questions, we compared whole-genome methylation profiles between ZBPS and manually selected spermatozoa (MSS) using single-cell genome-wide bisulfite sequencing. We found significant global DNA methylation differences. We also found that autism candidate genes had a greater number of differentially methylated regions (DMRs), due to the high proportion of autism genes with bivalent chromatin structures.

## Materials and Methods

### Semen Collection, Routine Semen Analysis, and Sperm Purification

Semen samples were collected from four healthy, fertile men. After retrieval, the semen samples were liquefied at a constant temperature of 37°C for 30–60min. After liquefaction, sperm parameters were determined using a computer-assisted sperm analysis system (Sperm Class Analyzer; Microptic, Barcelona, Spain). The spermatozoa were purified by gradient centrifugation using 90% and 45%gradients (SpermGrad; Vitrolife, Kungsbacka, Sweden) at 300 × g for 20min.The resulting supernatant was removed, and the sperm pellets were washed twice with G-IVF medium(G-IVF; Vitrolife, Kungsbacka, Sweden). Finally, the spermatozoa were cultured in G-IVF medium at a concentration of approximately 10 × 10^6^/mL.

### Sperm Selection

Immature oocytes were donated by couples who underwent ICSI. To collect ZPBS, approximately 10,000 spermatozoa were co-incubated with two or three immature oocytes in a50 µL G-IVF microdroplet covered with oil in an incubator with 6% CO_2_ for 2 hours. These oocytes were then washed in clear G-IVF microdroplets to remove loosely adhered spermatozoa. Sequentially, 30-50 spermatozoa bound to the ZP were collected using a micro-injection needle (Sunlight Medical, Jacksonville, FL, USA), and transferred to a PCR tube containing 10 µL of lysis buffer. To collect the MSS, another fraction of spermatozoa (10,000) was incubated under the same conditions as the ZPBS for 2 hours. Then, 30-50 spermatozoa in the microdroplet were collected by an embryologist trained to perform ICSI using a micro-needle under a microscope at 400× magnification (ZEISS, Jena, Germany), and transferred to a PCR tube containing 10 µL of lysis buffer. Thus, paired ZPBS and MSS samples were collected from one donor (e.g. ZPBS1 and MSS1 were recovered from donor1).

### Single-Cell Bisulfite Sequencing

Single-cell bisulfite sequencing (scBS-seq) was performed by Annoroad Gene Tech. Co., Ltd. (Beijing, China). The single-cell library was prepared as previously described using the following steps: bisulfite conversion, first-strand DNA synthesis, first-strand DNA purification, second-strand DNA synthesis, PCR amplification, library quantification, and sequencing (16). The sequencing data have been deposited in the SRA (https://www.ncbi.nlm.nih.gov/sra) under accession number PRJNA762253.

### Data Analysis

Raw reads were trimmed using Trimmomatic (v0.36, http://www.usadellab.org/cms/?page=trimmomatic) to remove the first nine base pairs, contaminating adapter sequences, poor quality read, and the bases at both ends and to discard reads with a length < 36nt (17). Clean reads were mapped to the human genome (Homo_sapiens. GRCh38.87v2) using Bismark (v0.16.3, https://www.bioinformatics.babraham.ac.uk/projects/bismark/) (18). We converted residual Gs in the reads to As and Cs to Ts *in silico* for the reads and the reference genome. We mapped both strands of the reads and the reference genome to each other and chose the best mapped read from four aligned results. We used uniquely mapped reads to calculate methylation rates.

Global methylation rates were calculated as the total number of methylated CpGs divided by the total number of covered CpGs × 100%.Global methylation rates were compared between ZPBS and MSS using Student’s t-test. For cluster analysis, we first searched the shared CpGs among all the samples and calculated the methylation rate for each shared CpG. We then constructed the methylation level clusters for these CpGs. For correlation analysis (Pearson’s), we used 2kb sliding windows to calculate the methylation rate among the whole genome and calculated Pearson’s correlation coefficient between samples. Mean correlations were compared between ZPBS and MSS using Student’s t-test. DMRs were identified using the Dispersion Shrinkage for Sequencing data (DSS) with the Wald test, based on the β-binomial distribution model. DMRs were defined as regions containing at least three CpGs, with a methylation rate difference between groups >0.2 and a *q*-value (Bonferroni-corrected P-value) <0.05 by Wald test. The proportion of genes with a DMR were compared by the chi-square test. Gene ontology (GO) analysis of the DMR-associated genes was performed using the Gene Ontology Resource (http://geneontology.org/). Functional elements, including promoters, exons, introns, and untranslated regions (UTRs), were determined using gene transfer format (GTF) files of the reference genome. False discovery rate (FDR)was calculated for each term and terms with FDR <0.05 were considered as significantly enriched. The promoters were defined as regions 2,000 bp upstream of the transcriptional start site. Methylation rate of a functional element was calculated as the number of methylated CpGs in the element divided by the total number of covered CpGs × 100% in this element. Comparisons of the proportion of DMRs among sub-groups at different elements were performed by the chi-square test. The comparisons of methylation levels of DMEs and standard deviations (SDs)between sub-groups were performed using a nonparametric test. A *P* value <0.05 was considered statistically significant. SPSS 19.0 (SPSS Inc., Chicago, IL, USA) and R (19) were used for data analysis.

Autism candidate genes were downloaded from the SFARI database (https://gene.sfari.org/database/human-gene/). Autism genes are significantly longer than random background genes with more introns and exons, which have a great influence to DMR rate. To avoid this bias, a three-to-one nearest neighbor caliper matching method without replacement was used to match the numbers of mapped introns and exons between the background and autism genes with a 0.2 SD caliper. Only protein-coding genes were included in the matching.

## Results

Sperm samples were donated by four men at the ages between 29 and 31. All of these men had normal semen parameters according to the WHO guidelines (2010) (**Table 1**).The genome-wide methylation level of each ZPBS and MSS sample with 30-50 spermatozoa was detected by bisulfite sequencing. There were 104 to 152 million clean reads per sample, and more than 50% of the clean reads were mapped to the reference genome (**Supplementary Table 1**). For each sample, 45.6% to 48.6% of the CpGs in the genome were covered, and about 30% CpGs were covered twice or more times (**Supplementary Table 2**). The average of mean coverage for covered CpGs was 2.6.Sample purity was tested by calculating the methylation levels of imprinted genes. As expected, imprinting control region 1 (ICR1) of the paternally imprinted gene,*H19*, was hyper-methylated, while the DMR of the maternally imprinted gene, *MEST*, was hypo-methylated in all samples (**Supplementary Figure 1**). There were no significant differences in the methylation rates of ICR1 (ZPBS *vs* MSS: 95.0 ± 2.2% *vs* 89.9 ± 4.8%, *P*>0.05) or *MEST* (ZPBS *vs* MSS: 3.4 ± 1.7% *vs* 2.4 ± 1.3%, *P*>0.05) between groups.

**Table 1:** Donor characteristics.

Donor	Age (y)	BMI (km/m^2^)	Volume(mL)	Sperm concentration (x10^6^/mL)	Sperm motility(%)	PR(%)
1	31	20.8	2.3	98.3	77.7	41.9
2	30	19.5	2	134.7	85.7	57.3
3	29	20.8	6.7	43.3	74	49.7
4	29	19.6	6.4	164.7	79.8	42.4

BMI, body mass index; PR, progressive motility rate.

**Figure 1:**
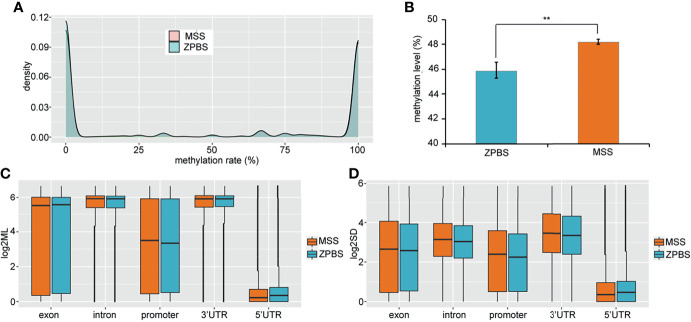
The genome-wide methylation pattern of ZPBS and MSS. **(A)** The distribution of methylation levels in shared CpGs among samples. **(B)** A comparison of global methylation levels between ZPBS and MSS. **(C)** A comparison of methylation rates in different functional elements. **(D)** A comparison of standard deviations of element methylation levels among samples between ZPBS and MSS.**< 0.01.

To compare the DNA methylation profiles of different samples, the methylation rates of CpGs covered in all eight samples were calculated and clustered. The results showed that the ZPBS and MSS from the same ejaculate clustered together (**Supplementary Figure 2A**). We also compared the correlations between each sample for the eight samples and found that the correlation coefficient was slightly higher among ZPBS samples than MSS samples (0.822*vs*0.807, *P*=0.045, **Supplementary Figure 2B**). As a feature of single-cell methylation sequencing, the methylation rates of most CpGs were either 0% or 100% (**Figure 1A**). The global methylation levels of ZPBS were significantly lower than those of MSS (**Figure 1B**). We further compared the methylation levels of different functional elements. The introns and 3′-UTRs were hyper-methylated while the 5′-UTRs were hypo-methylated (**Figure 1C**). The methylation levels of the exons and promoters were diverse, with wide ranges (**Figure 1C**). To compare inter-sample heterogeneity, we calculated the SDs of each element for ZPBS and MSS samples. The SDs in ZPBS were lower in exons, introns, promoters, and 3′-UTRs but not 5′-UTRs, suggesting lower inter-sample heterogeneity in most elements of ZPBS compared with those of MSS (**Figure 1D**).

**Figure 2:**
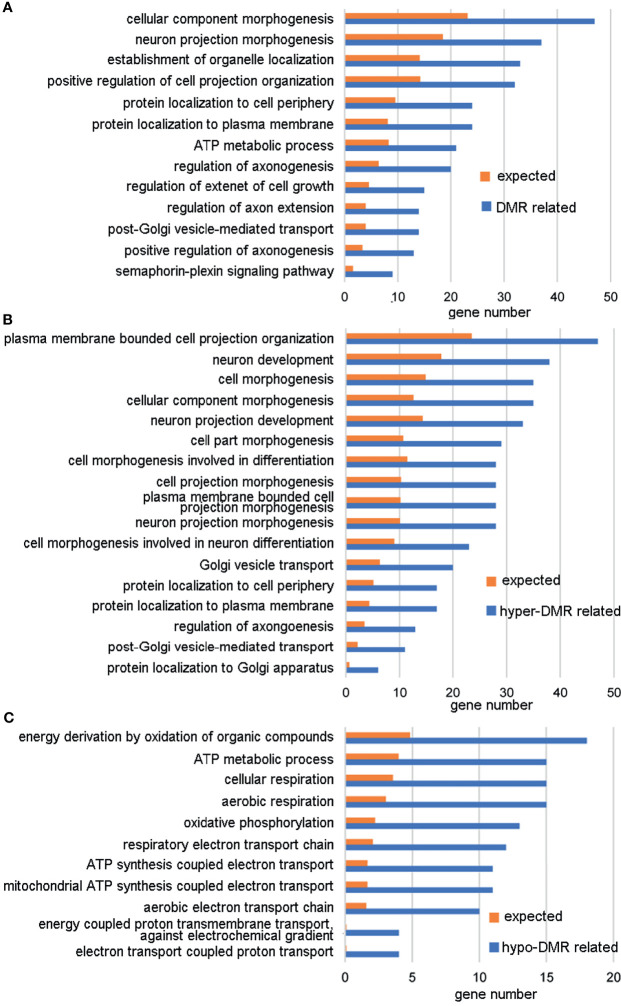
GO analysis of genes with DMRs in promoters. **(A)** GO terms with enrichment ≥2 and FDR < 0.05 for genes with hyper-DMRs and/or hypo-DMRs in promoters. **(B)** GO terms with enrichment ≥2 and FDR < 0.05 for genes with hyper-DMRs in promoters. **(C)** GO terms with enrichment ≥ 2 and FDR < 0.05 for genes with hypo-DMRs in promoters.

A DMR was defined as a region including at least three CpGs with significantly different DNA methylation levels between the ZPBS and MSS groups. There were 11,175 DMRs identified throughout the whole genome, 52.3% of which were hypo-methylated in ZPBS (**Supplementary Table 3**). A DMR-related gene was defined as one gene overlapping with at least one DMR in its promoter and/or gene body. Of 7,641 DMR-related genes, 5,656 genes were annotated for biological processes in a GO analysis. The top five enriched GO terms were anterior/posterior axon guidance (GO:0033564), cerebrospinal fluid secretion (GO:0033326), synaptic transmission, glycinergic (GO:0060012), transmembrane receptor protein tyrosine phosphatase signaling pathway (GO:0007185), and semaphorin-plexin signaling pathway involved in neuron projection guidance (GO:1902285). These five biological processes included 32 DMR-associated genes (**Supplementary Table 4**), of which seven genes, including *TRIO*(high confidence),*DCC*(strong candidate), *PLXNA4*(strong candidate), *PLXNB1*(strong candidate), *GLRA2*(suggestive evidence), *NRP2*(suggestive evidence), and *PLXNA3*(suggestive evidence), were autism candidate genes reported in the SFARI database (https://gene.sfari.org/database/human-gene/).To avoid enrichment biases derived by gene length, genes with DMRs in promoters were analyzed by GO. These genes were also enriched in biological processes involved in neural development (**Figure 2A**). Then, genes with ZPBS-hyper-methylated DMRs and ZPBS-hypo-methylated DMRs in promoter were enriched separately. Interestingly, hyper-DMR related genes were enriched in terms related with neural development (**Figure 2B**), while hypo-DMR related genes were enriched in terms involving in ATP synthesis (**Figure 2C**).

**Figure 3:**
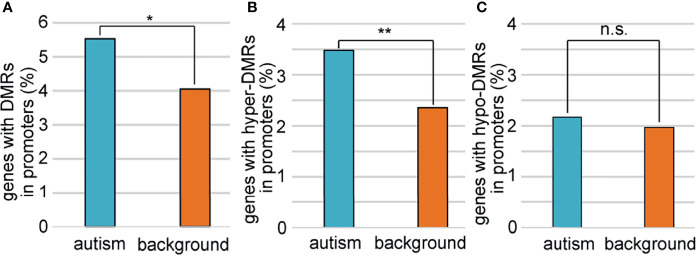
Comparisons of genes with DMRs in promoters between autism and background genes. **(A)** The proportion of genes with DMRs in promoters was higher among autism genes than among background genes. **(B)**The proportions of genes with hyper-DMRs in promoter were higher among autism genes than among background genes. **(C)** The proportions of genes with hypo-DMRs in promoter were similar among autism genes with among background genes. *P < 0.05; **P < 0.01; n.s., not significant.

To explore the relationship between sperm selection and the risk of autism in children conceived by ICSI, we analyzed the methylation status of 1,003 autism candidate genes reported in the SFARI database (**Supplementary Table 5**). We first compared the methylation profiles of autism genes between ZPBS and MSS, and found that the methylation rates of ZPBS functional elements showed more centralized profiles at about 68% compared with MSS (**Supplementary Figure 3**), indicating a lower methylation variability in ZPBS. Then, we compared the proportions of DMR-related genes between autism and background genes. We found more autism genes with DMRs in their promoter (**Figure 3A**). In accordance with GO analysis, there were more promoters overlapping with hyper-DMRs (**Figure 3B**), but not hypo-DMRs (**Figure 3C**), in autism genes compared with background genes.

**Figure 4:**
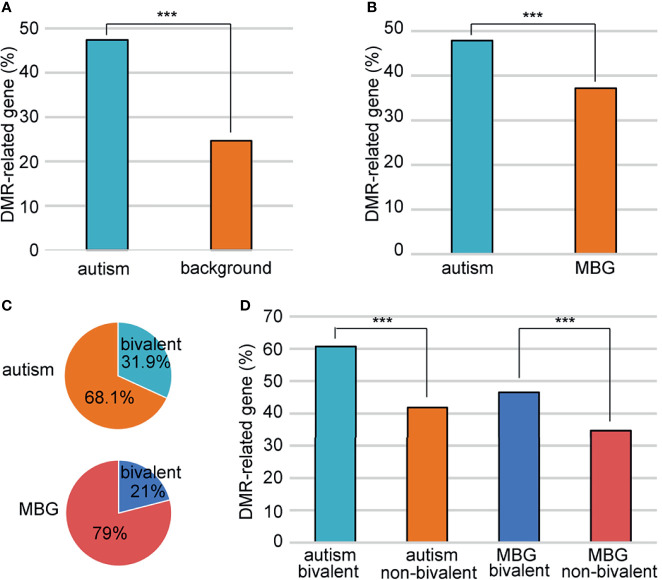
Association of the bivalent chromatin structure and high DMR rates. **(A)** A comparison of DMR-related gene rate between autism and background genes. **(B)** A comparison of DMR-related gene rate between autism and matched background genes (MBG). **(C)** Bivalent chromatin structure was more common in autism genes than in background genes. **(D)** Bivalent chromatin structure was associated with a higher frequency of DMRs. MBG, matched background genes; ***P < 0.001.

Just like genes with DMRs in promoters, We also found more genes with DMRs in promoters and/or gene bodies in autism candidates than background genes (**Figure 4A**). However, as autism genes were significantly longer than random genes, there were more introns and exons in autism genes, which would great influence the number of DMRs. To avoid this bias, we defined a group of background genes with matched number of introns and exons (**Supplementary Table 6**). A total of2806 background protein-coding genes were matched to 951autism genes. The rate of DMR-related genes was still higher in these autism genes (47.8%) than matched background genes (37.1%) (**Figure 4B**).

Schrott et al. (20) showed that autism candidate genes are significantly enriched for bivalent chromatin structures, which makes the methylation of these genes vulnerable to environmental exposure. We sought to determine whether this bivalent structure also contributed to the higher DMR rate in autism genes between ZPBS and MSS. Indeed, there was a higher proportion of genes with bivalent chromatin (21) among autism genes than among matched background genes (**Figure 4C**). Moreover, the proportion of genes with DMRs was higher among genes with bivalent structures, both for autism candidate genes and matched background genes (**Figure 4D**).

## Discussion

The spermatozoa are a group of specialized cells with high heterogeneity. DNA methylation levels vary between different ejaculates from the same man and between high- and low-quality fractions of the same ejaculate (14). The morphological or functional differences between spermatozoa from the same ejaculate may be due to epigenetic factors. We compared the genome-wide DNA methylation profiles between ZPBS and MSS and found global methylation differences and differences at a number of specific genes. We also found that these methylation differences were enriched in autism genes, which provides an explanation for the higher rate of autism in offspring conceived *via* ICSI.

During spermatogenesis, the genome undergoes demethylation and *de novo* methylation (22). Aberrant methylation during this process affects gene expression, imprinting, protamine transition, and chromosome structure, leading to abnormal sperm count, motility, and morphology (23). Several studies have linked aberrant DNA methylation in sperm to a decrease in semen parameters. Houshdaran et al. (24) found that sperm DNA is hyper-methylated in samples with poor sperm concentration, motility, and morphology. Sujit et al. (25) reported that the sperm DNA methylation levels of spermatogenesis-related genes are higher in men with oligozoospermia than in men with normal semen parameters. These results are consistent with our findings that global DNA methylation levels were higher in MSS than in ZPBS (Fig. 1B). In addition, genes with ZPBS-hypo-DMRs in promoters were significantly enriched in processes involving in ATP synthesis which was essential for sperm motility, acrosin activity, acrosome reaction capability and chromatin integrity (26–28).Thus, DNA hyper-methylation in sperm may affect energy synthesis leading to reduced sperm fertilization ability.

Several studies have suggested that children born through ICSI have an increased risk of autism and intellectual disability, which may be influenced by paternal characteristics linked to male infertility or the processes of ICSI treatment (4, 5, 29, 30). Kissin et al. (5) reported a higher incidence of autism in children conceived after ICSI compared with those conceived *via* conventional IVF in a cohort study of 42,383 children conceived with assisted reproductive technology (1997–2006). Importantly, they further reported that the association between autism and ICSI was stronger among children conceived with ICSI for non-male factors, suggesting that a diagnosis of male infertility was not associated with an increased risk of autism. These results may be explained by the hypothesis that the bypassing of the natural sperm selection process during ICSI may result in the injection of a spermatozoon carrying genetic or epigenetic mutations related to autism, leading to an increased risk of autism. Our study provided evidence supporting this hypothesis. On one hand, the proportion of autism candidate genes with at least one DMR between ZPBS and MSS was significantly higher than the corresponding proportion among matched background genes (**Figure 4B**). On the other hand, genes with DMRs in promoter were enriched in functions related to neuron projection, axonogenesis, axon extension, semaphoring-plexin signaling pathway (**Figure 2**). These processes are all closely associated with the prevalence of autism.

Few studies have linked DNA methylation alterations in autism candidate genes with sperm selection during fertilization. However, there is evidence for a relationship between autism and locus-specific and genome-wide changes in DNA methylation. Garrido et al. (31) compared genome-wide methylation levels between sperm samples obtained from fathers of children with and without autism and found that genes associated with the DMRs are linked to previously known autism spectrum disorder genes and other neurobiology-related genes. Advanced paternal age has been suggested as a risk factor for autism (32). Global or locus-specific DNA methylation levels are reported to increase in sperm as men age (33). Some of the age-related changes in sperm DNA methylation occur at genes associated with neuropsychiatric disorders (33).

The prevalence of autism is increasing. In the United States, the prevalence reported by the Centers for Disease Control and Prevention was 1 in 150 in 2000, but reached 1 in 54 in 2016 (https://www.cdc.gov/ncbddd/autism/data.html). Environmental factors are thought to contribute to the increased prevalence of autism. Epigenetic factors play an important role in bridging the environment and disease. Two studies from one group reported that the methylation of autism candidate genes in rat sperm is affected by environmental exposure to tetrahydrocannabinol and nicotine (20, 34). They also discovered that autism candidate genes are significantly enriched for bivalent chromatin structure and proposed that this configuration may increase the vulnerability of genes in sperm to disrupted methylation (20). In the current study, a higher rate of DMRs between MSS and ZPBS was found in autism genes than in matched background genes, suggesting higher DNA methylation variability of autism genes in sperm (**Figure 4B**). This may be the result of special methylation features of autism genes. Indeed, we found that the number of DMRs was higher in autism candidate genes with a bivalent structure (**Figure 4D**).

The above findings provide insights into the reason for the association between ICSI and the risk of autism in offspring. The methylation of autism candidate genes in sperm is vulnerable because of the unique bivalent chromatin structure. This susceptibility of autism genes leads to higher DNA methylation heterogeneity between spermatozoa. Spermatozoa selected by an embryologist for ICSI escape the natural selection process and have a higher risk of carrying more DNA methylation alterations in autism candidate genes, resulting in abnormal expression of these genes and eventually leading to a higher risk of autism in offspring conceived by ICSI using these spermatozoa.

To the best of our knowledge, this is the first study to compare methylation levels between ZPBS and MSS. Using scBS-seq, approximately half of all CpGs were detected. However, due to the limited number of ZPBS samples and the nature of scBS-seq, the sequencing depth was limited. The mean coverage for covered CpGs was only 2.6. Therefore, intermediate methylation information was missing for a lot of single CpGs. However, the methylation levels of most CpGs in spermatozoa have been reported to be either 0 or 100% (35, 36), which may eliminate the potential bias introduced by this sequencing technique.

The global methylation levels in current study were about 46-48% which was similar with those in previous studies using Infinium Human Methylation beadchip and reduced representation bisulfite sequencing (RRBS) (35, 36). But comparing with whole genome bisulfite sequencing (WGBS), the global methylation levels in this study were much lower, which may due to the uneven distribution of reads (35, 37). It has been reported that the enrichment towards exons, promoters and CGIs is exaggerated in scBS-Seq libraries (38). Promoters and CGIs are hypo-methylated in sperm (39). Chan et al. (35) have compared methylation levels of sperm using WGBS between two studies, and found that the average methylation level was lower (69.4% *vs* 80.5%) in the study (WGBS-Prev) with lower coverage rate. This result was explained by the fact that a greater proportion of highly covered sites from the WGBS-Prev was found within promoter-transcriptional start site (TSS) regions as well as within CpG islands.

In summary, this study compared the methylation profiles of ZP-bound and manually selected spermatozoa and found differential methylation levels globally and at specific loci. DMR-related genes were enriched in biological processes related to the onset of autism. The frequency DMRs was higher in autism genes as a result of the specific bivalent structure of these genes. Our results provide an explanation for the higher risk of autism in offspring conceived by ICSI, and suggest that it should be taken seriously in selecting ICSI over conventional IVF for non-male factor infertility during assisted reproduction technology.

## Data Availability Statement

The datasets presented in this study can be found in online repositories. The names of the repository/repositories and accession number(s) can be found in the article/**Supplementary Material**.

## Ethics Statement

The studies involving human participants were reviewed and approved by Ethics Committee of the First Affiliated Hospital of Kunming Medical University. The patients/participants provided their written informed consent to participate in this study.

## Author Contributions

LW and MC conducted the research. All authors provided contributions to analysis and interpretation of data. SZ designed and supervised the study, and drafted the manuscript. LW and MC contributed equally as corresponding authors. All authors contributed to the article and approved the submitted version.

## Funding

This work was supported by the Natural National Science Foundation of China, under grant number 81760269, the Yunnan Provincial Science and Technology Department, under grant numbers 2018FB126 and 2017HB046, and the Health Commission of Yunnan Province, under grant number D-2017021. The open access publication fees were supported by the First Affiliated Hospital of Kunming Medical University.

## Conflict of Interest

The authors declare that the research was conducted in the absence of any commercial or financial relationships that could be construed as a potential conflict of interest.

## Publisher’s Note

All claims expressed in this article are solely those of the authors and do not necessarily represent those of their affiliated organizations, or those of the publisher, the editors and the reviewers. Any product that may be evaluated in this article, or claim that may be made by its manufacturer, is not guaranteed or endorsed by the publisher.
