# Supplementary material for: DNA Methylation Differences Between Zona Pellucida-Bound and Manually Selected Spermatozoa Are Associated With Autism Susceptibility
Source: Front Endocrinol (Lausanne). 2021 Nov 9;12:774260. doi: 10.3389/fendo.2021.774260 (PMC8630694; doi:10.3389/fendo.2021.774260)
Supplement: Supplementary file 1 [file DataSheet_1.pdf]

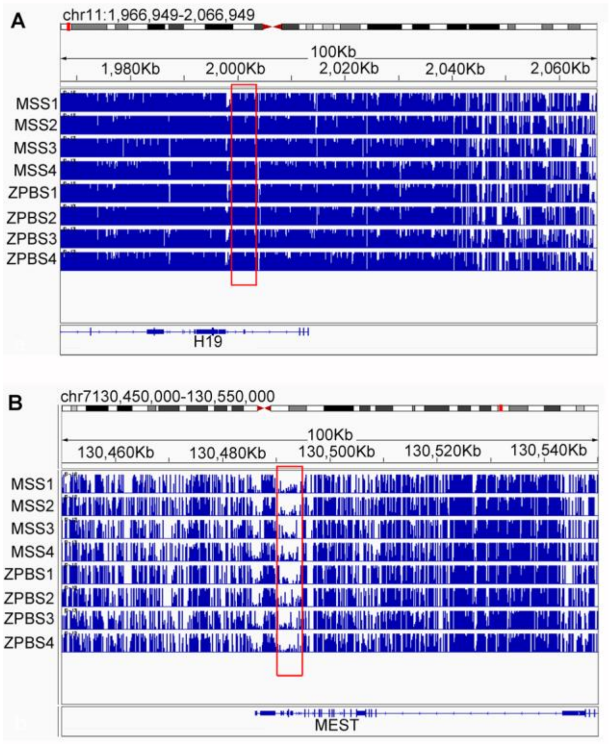

Supplementary Figure 1. Integrative genomics viewer snapshot of the methylation of the paternally imprinted gene, *H19*, and the maternally imprinted gene, *MEST*. (A) *H19* imprinting control region 1, indicated by the red box, was hyper-methylated in all samples. (B) The differentially methylated region of *MEST*, indicated by the red box, was hypo-methylated in all samples.
