# Supplementary material for: DNA Methylation Differences Between Zona Pellucida-Bound and Manually Selected Spermatozoa Are Associated With Autism Susceptibility
Source: Front Endocrinol (Lausanne). 2021 Nov 9;12:774260. doi: 10.3389/fendo.2021.774260 (PMC8630694; doi:10.3389/fendo.2021.774260)
Supplement: Supplementary file 2 [file DataSheet_2.pdf]

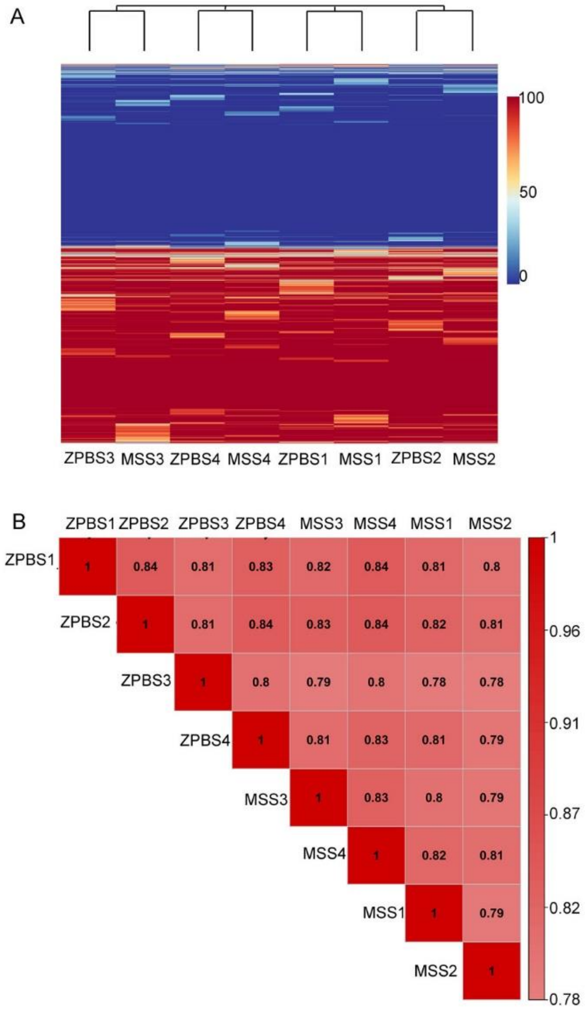

Supplementary Figure 2. Clustering and correlation analyses of samples based on CpG methylation level. (A) Hierarchical clustering by CpG methylation levels in different sperm samples. Unmethylated CpGs are indicated in blue, and completely methylated CpGs are indicated in red. Each column represents one sample. ZPBS and MSS samples from the same ejaculate are clustered together. (B) A heatmap of Pearson's correlation coefficients for global DNA methylation profiles in different samples. The color scale from pink to red represents correlation coefficients from 0.78 to 1.0.
