# Supplementary material for: DNA Methylation Differences Between Zona Pellucida-Bound and Manually Selected Spermatozoa Are Associated With Autism Susceptibility
Source: Front Endocrinol (Lausanne). 2021 Nov 9;12:774260. doi: 10.3389/fendo.2021.774260 (PMC8630694; doi:10.3389/fendo.2021.774260)
Supplement: Supplementary file 3 [file DataSheet_3.pdf]

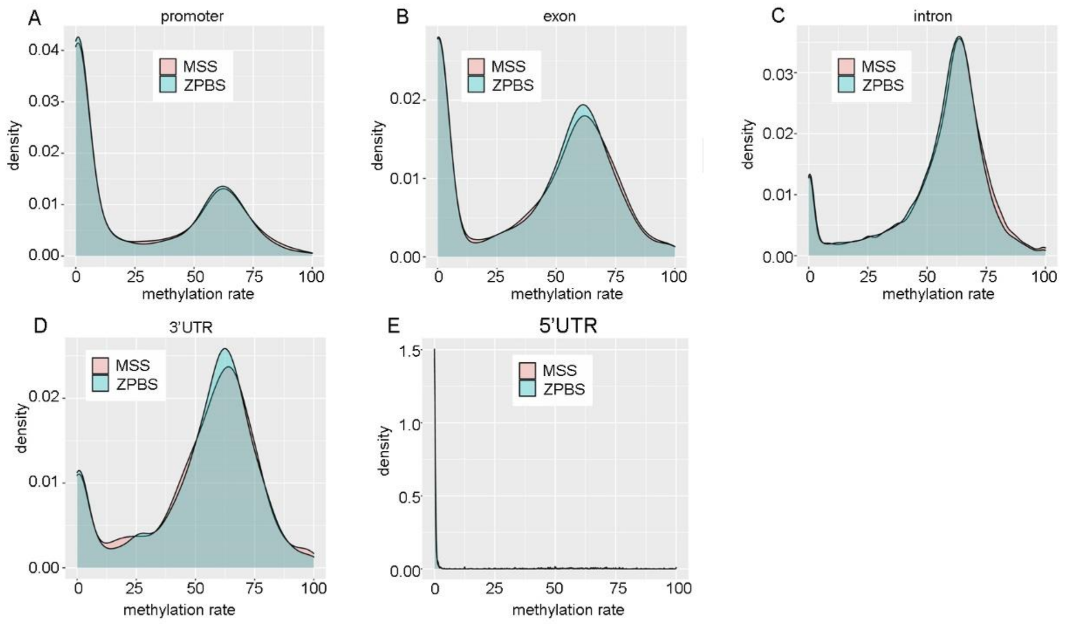

Supplementary Figure 3. Comparisons for methylation rate distributions of autism genes between ZPBS and MSS. Methylation rates of promoter (A), exon (B), intron (C), 3'UTR (D) and 5'UTR (E) were compared between ZPBS and MSS.
