# Supplementary material for: DNA Methylation Differences Between Zona Pellucida-Bound and Manually Selected Spermatozoa Are Associated With Autism Susceptibility
Source: Front Endocrinol (Lausanne). 2021 Nov 9;12:774260. doi: 10.3389/fendo.2021.774260 (PMC8630694; doi:10.3389/fendo.2021.774260)
Supplement: Supplementary file 4 [file Table_1.docx]

Supplementary Table 1 mapped read statistics.

| Sample | ZPBS1 | ZPBS2 | ZPBS3 | ZPBS4 | MSS1 | MSS2 | MSS3 | MSS4 |
| --- | --- | --- | --- | --- | --- | --- | --- | --- |
| Clean Reads | 128,139,714 | 104,411,532 | 152,324,626 | 131,490,728 | 113,361,188 | 105,988,202 | 128,257,504 | 150,571,640 |
| Mapped Reads | 71,218,110 | 60,952,448 | 81,114,561 | 73,396,208 | 63,335,000 | 57,720,479 | 64,899,811 | 75,406,374 |
| Mapped Ratio (%) | 55.58 | 58.38 | 53.25 | 55.82 | 55.87 | 54.46 | 50.6 | 50.08 |
| Unique Mapped Reads | 64,830,302 | 56,265,966 | 72,969,264 | 66,559,179 | 57,850,973 | 53,500,897 | 59,703,666 | 68,683,326 |
| Unique Mapped Ratio (%) | 50.59 | 53.89 | 47.9 | 50.62 | 51.03 | 50.48 | 46.55 | 45.62 |
| Insert Size (bp) | 240.74 | 282.31 | 200.62 | 228.05 | 251.61 | 272.59 | 229.92 | 214.46 |
| Duplication (%) | 7.42 | 8.44 | 8.12 | 7.35 | 9.37 | 8.72 | 7.43 | 8.27 |
