# Supplementary material for: DNA Methylation Differences Between Zona Pellucida-Bound and Manually Selected Spermatozoa Are Associated With Autism Susceptibility
Source: Front Endocrinol (Lausanne). 2021 Nov 9;12:774260. doi: 10.3389/fendo.2021.774260 (PMC8630694; doi:10.3389/fendo.2021.774260)
Supplement: Supplementary file 5 [file Table_2.docx]

Supplementary Table 2 CpG coverages of each sample.

| Sample | ZPBS1 | ZPBS2 | ZPBS3 | ZPBS4 | MSS1 | MSS2 | MSS3 | MSS4 |
| --- | --- | --- | --- | --- | --- | --- | --- | --- |
| CpGs (1x) | 28,533,752 | 27,943,757 | 28,227,386 | 28,420,053 | 27,138,028 | 26,790,533 | 26,739,048 | 27,255,602 |
| Mean Coverage (1x) | 2.659 | 2.2999 | 3.0201 | 2.7388 | 2.4038 | 2.2823 | 2.5197 | 2.7615 |
| Percentage (1x) (%) | 48.6172 | 47.6119 | 48.0952 | 48.4235 | 46.2391 | 45.647 | 45.5593 | 46.4394 |
| CpGs (2x) | 18,333,595 | 16,486,978 | 19,357,025 | 18,527,713 | 16,298,101 | 15,424,136 | 16,475,449 | 17,687,089 |
| Mean Coverage (2x) | 3.5821 | 3.2031 | 3.9459 | 3.6672 | 3.3374 | 3.2272 | 3.4663 | 3.7145 |
| Percentage (2x) (%) | 31.2377 | 28.0913 | 32.9814 | 31.5684 | 27.7695 | 26.2804 | 28.0717 | 30.1361 |
| CpGs (3x) | 11,348,999 | 9,171,515 | 12,720,320 | 11,652,658 | 9,387,862 | 8,519,313 | 9,840,405 | 11,186,275 |
| Mean Coverage (3x) | 4.5557 | 4.1628 | 4.9611 | 4.6508 | 4.3219 | 4.2218 | 4.4551 | 4.7109 |
| Percentage (3x) (%) | 19.337 | 15.6269 | 21.6735 | 19.8544 | 15.9955 | 14.5156 | 16.7666 | 19.0597 |
| CpGs (4x) | 6,914,083 | 4,925,630 | 8,395,386 | 7,252,429 | 5,298,018 | 4,616,284 | 5,807,256 | 7,048,940 |
| Mean Coverage (4x) | 5.5536 | 5.1651 | 5.9714 | 5.6524 | 5.3423 | 5.2549 | 5.4656 | 5.7151 |
| Percentage (4x) (%) | 11.7805 | 8.3925 | 14.3045 | 12.357 | 9.027 | 7.8654 | 9.8947 | 12.0103 |
| CpGs (5x) | 4,159,210 | 2,580,881 | 5,493,597 | 4,467,283 | 2,951,731 | 2,477,453 | 3,396,591 | 4,407,740 |
| Mean Coverage (5x) | 6.5826 | 6.2236 | 7.0127 | 6.6825 | 6.4093 | 6.3383 | 6.5058 | 6.7428 |
| Percentage (5x) (%) | 7.0867 | 4.3974 | 9.3603 | 7.6116 | 5.0293 | 4.2212 | 5.7873 | 7.5101 |
| CpGs (10x) | 302,012 | 92,771 | 654,124 | 362,979 | 164,403 | 120,558 | 228,959 | 405,577 |
| Mean Coverage (10x) | 13.1547 | 15.974 | 12.9528 | 13.2104 | 14.0212 | 14.6274 | 13.114 | 12.7709 |
| Percentage (10x) (%) | 0.5146 | 0.1581 | 1.1145 | 0.6185 | 0.2801 | 0.2054 | 0.3901 | 0.691 |
