# Supplementary material for: DNA Methylation Differences Between Zona Pellucida-Bound and Manually Selected Spermatozoa Are Associated With Autism Susceptibility
Source: Front Endocrinol (Lausanne). 2021 Nov 9;12:774260. doi: 10.3389/fendo.2021.774260 (PMC8630694; doi:10.3389/fendo.2021.774260)
Supplement: Supplementary file 7 [file Table_4.docx]

Supplementary Table 4 Top five enriched biology processes.

| GO ID | GO term | Gene ID | gene name | description | autism candidate |
| --- | --- | --- | --- | --- | --- |
| GO:0033564 | anterior/posterior axon guidance | ENSG00000113763 | UNC5A | unc-5 netrin receptor A |  |
|  |  | ENSG00000065320 | NTN1L | netrin 1 |  |
|  |  | ENSG00000187323 | DCC | DCC netrin 1 receptor | yes |
|  |  | ENSG00000107731 | UNC5B | unc-5 netrin receptor B |  |
|  |  | ENSG00000182168 | UNC5C | unc-5 netrin receptor C |  |
| GO:0033326 | cerebrospinal fluid secretion | ENSG00000188687 | SLC4A5 | solute carrier family 4 member 5 |  |
|  |  | ENSG00000240583 | AQP1 | aquaporin 1 |  |
|  |  | ENSG00000250424 |  | novel protein, MINDY4 and AQP1 readthrough |  |
|  |  | ENSG00000264324 |  | novel protein |  |
|  |  | ENSG00000109944 | JHY | junctional cadherin complex regulator |  |
| GO:0060012 | synaptic transmission, glycinergic | ENSG00000145451 | GLRA3 | glycine receptor alpha 3 |  |
|  |  | ENSG00000196517 | SLC6A5 | solute carrier family 6 member 9 |  |
|  |  | ENSG00000101958 | GLRA2 | glycine receptor alpha 2 | yes |
|  |  | ENSG00000109738 | GLRB | glycine receptor beta |  |
|  |  | ENSG00000165970 | SLC6A5 | solute carrier family 6 member 5 |  |
|  |  | ENSG00000188828 | GLRA4 | glycine receptor alpha-4 |  |
| GO:0007185 | transmembrane receptor protein tyrosine phosphatase signaling pathway | ENSG00000060656 | PTPRU | protein tyrosine phosphatase receptor type U |  |
|  |  | ENSG00000142949 | PTPRF | receptor-type tyrosine-protein phosphatase F |  |
|  |  | ENSG00000153707 | PTPRD | receptor-type tyrosine-protein phosphatase delta |  |
|  |  | ENSG00000166225 | FRS2 | fibroblast growth factor receptor substrate 2 |  |
|  |  | ENSG00000038382 | TRIO | trio Rho guanine nucleotide exchange factor | yes |
|  |  | ENSG00000225011 |  | protein tyrosine phosphatase, receptor type, U (PTPRU) pseudogene |  |
| GO:1902285 | semaphorin-plexin signaling pathway involved in neuron projection guidance | ENSG00000004399 | PLXND1 | plexin D1 |  |
|  |  | ENSG00000075213 | SEMA3A | semaphorin 3A |  |
|  |  | ENSG00000118257 | NRP2 | neuropilin 2 | yes |
|  |  | ENSG00000136040 | PLXNC1 | plexin C1 |  |
|  |  | ENSG00000221866 | PLXNA4 | plexin A4 | yes |
|  |  | ENSG00000001617 | SEMA3F | semaphorin 3F |  |
|  |  | ENSG00000076356 | PLXNA2 | plexin A2 |  |
|  |  | ENSG00000130827 | PLXNA3 | plexin A3 | yes |
|  |  | ENSG00000164050 | PLXNB1 | plexin B1 | yes |
